# Structural scoliosis secondary to thoracic osteoid osteoma: a case report of delayed diagnosis

**DOI:** 10.1007/s43390-022-00553-1

**Published:** 2022-08-16

**Authors:** Mohamed Zairi, Mohamed Nabil Nessib

**Affiliations:** Faculty of Medicine of Tunis, Department of Pediatric Orthopedic Surgery, Bechir Hamza Children’s Hospital, 167 Boulevard du 9 Avril 1938, Tunis, Tunisia

**Keywords:** Scoliosis, Osteoid steoma, Adolescent, Delayed diagnosis

## Abstract

**Purpose:**

The aim of this case report is to show that late diagnosis of vertebral osteoid osteoma gives rise to structural scoliosis which sometimes requires long-term management.

**Methods:**

We report a case of an osteoid osteoma in the thoracic spine associated with structural scoliosis. We describe a 14-year-old boy who complained chronic nightly left back pain and scoliosis. Spine’s X-ray was reported thoraco-lumber scoliosis without bone lesion.

**Results:**

MRI as well as technetium-99 m total body bone scan and a computed tomography scan revealed a bony lesion in the upper left joint of T11 vertebra consistent with the diagnosis of OO. Anatomopathological study of the resection piece confirmed the diagnosis of OO. Surgical excision of the tumor resolved pains, but scoliosis needed an orthopedic treatment for 1 year.

**Conclusion:**

Through this case, it has been demonstrated that late diagnosed vertebral OO can be the cause of structural scoliosis. Clinical and radiological results indicate that OO resection is an effective and safe method of treatment.

**Levels of evidence:**

IV.

## Introduction

The occurrence of scoliosis in older children and adolescents is most often idiopathic. Nevertheless, an etiology must be systematically ruled out in the face of the painful nature of scoliosis. The therapeutic strategy must be carried out according to a schedule taking into account two factors: scoliosis and its etiology. OO is one of the etiologies to think about.

## Case report

**Fig. 1:**
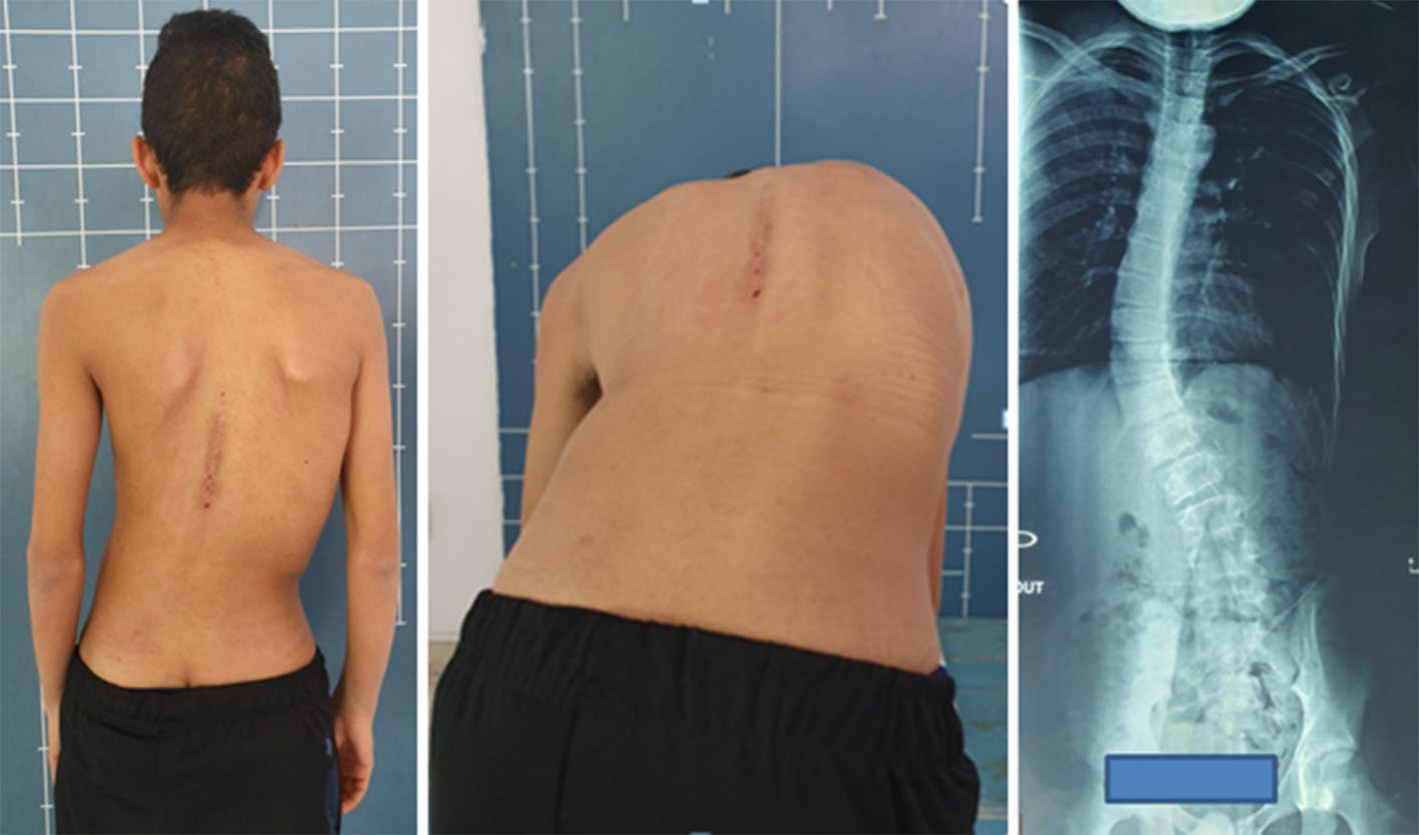
In standing position: deformation of the midline, leaning forward: straight gibbosity and the x-ray shows structural scoliosis

**Fig. 2:**
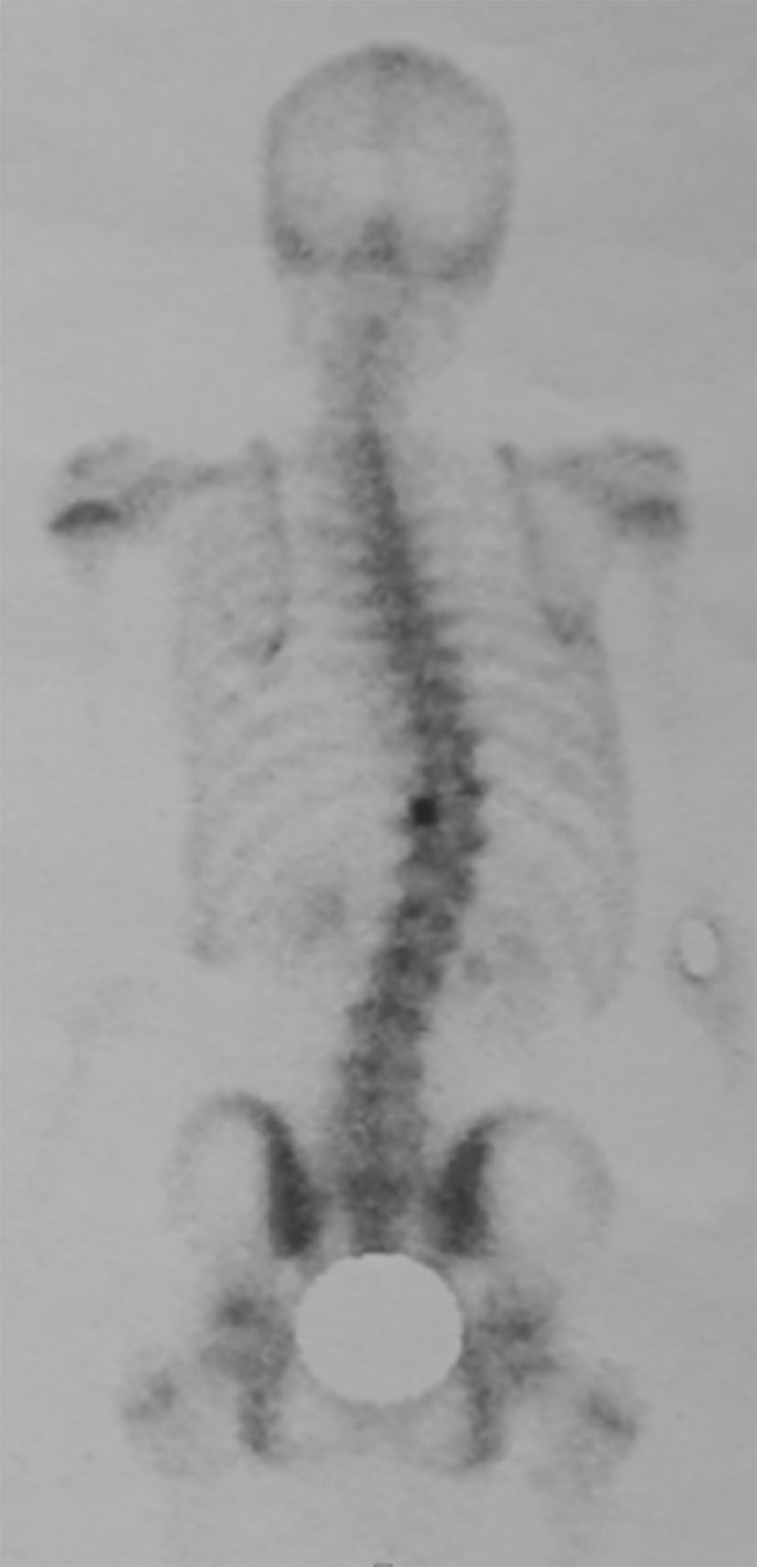
Bone scintigraphy: hyperfixation of the left superior articular process of T11

**Fig. 3:**
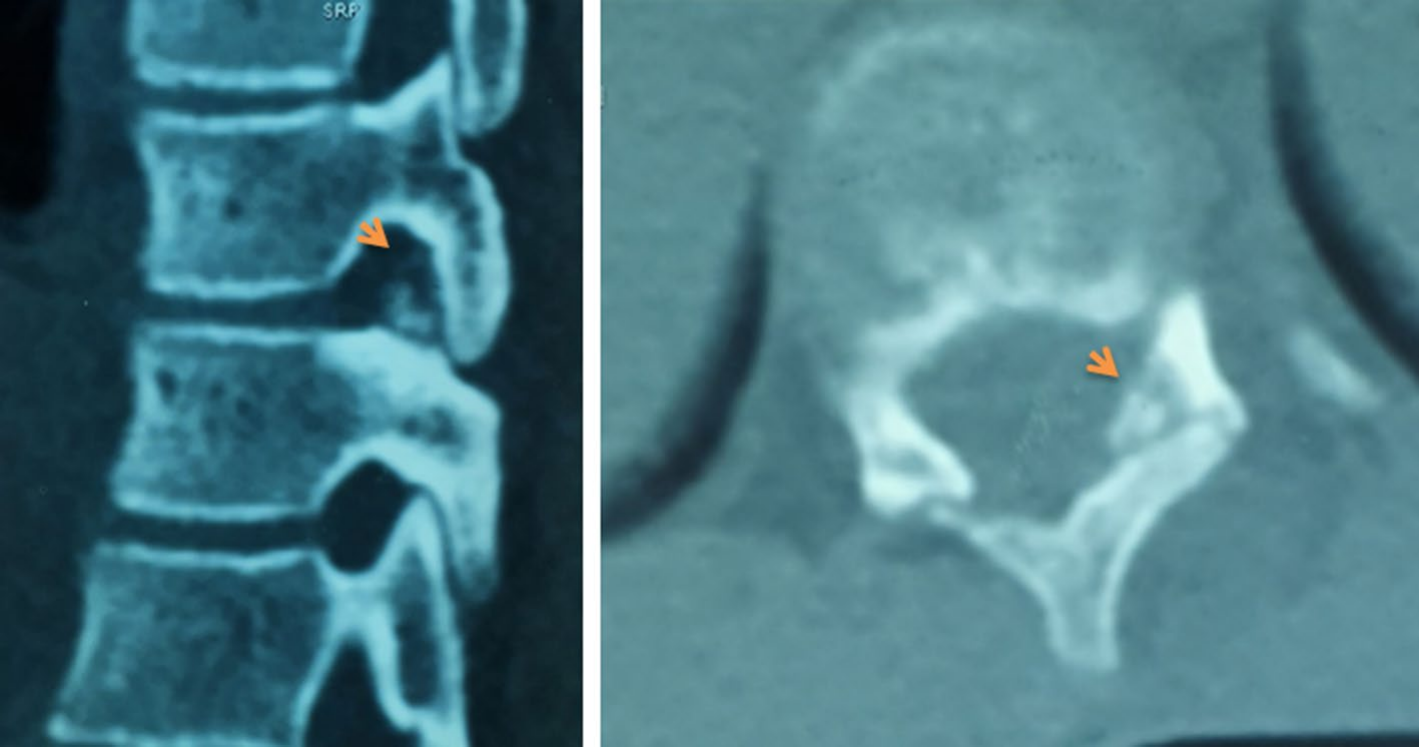
CT scan concluded with a rosette image with a central nidus suggestive of osteoid osteoma

**Fig. 4:**
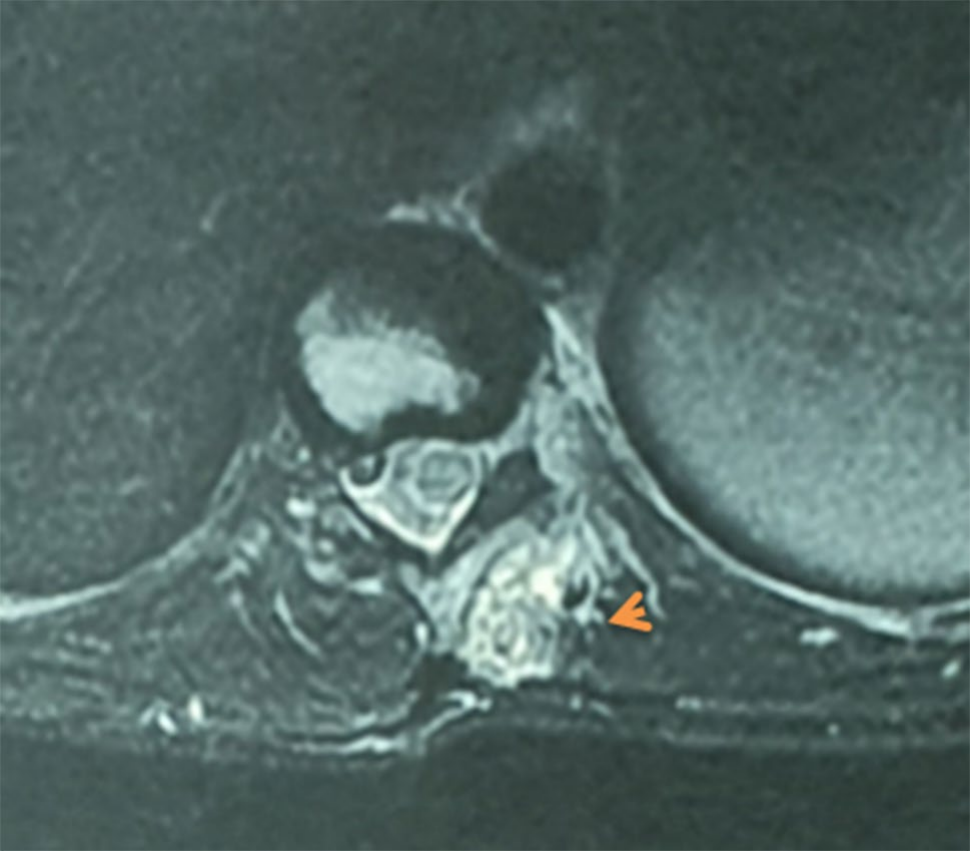
MRI showed signal abnormalities in the left superior articular process of T11, an intense response of peripheral soft tissues

**Fig. 5:**
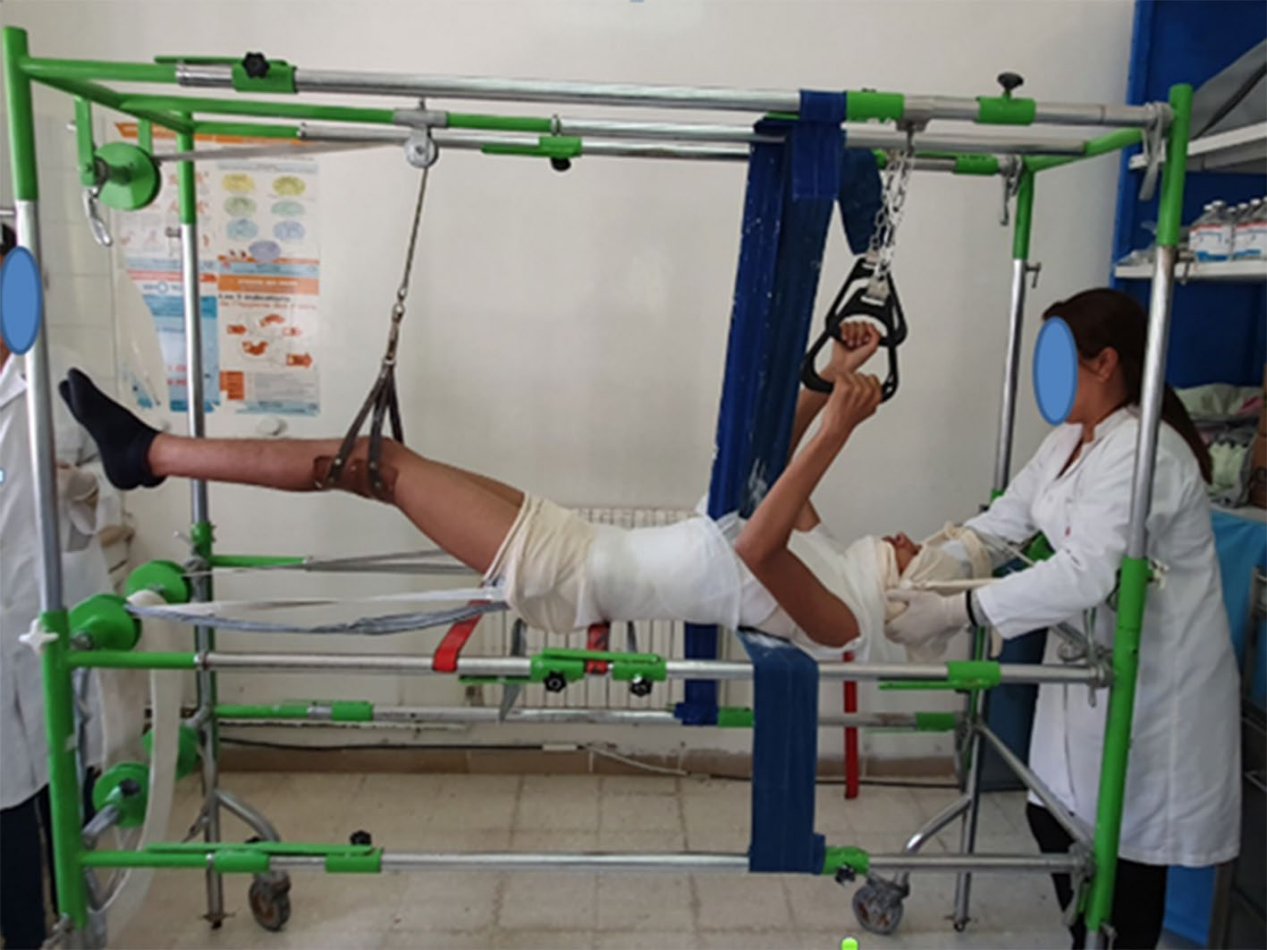
Cotrel traction: correction of scoliosis

**Fig. 6:**
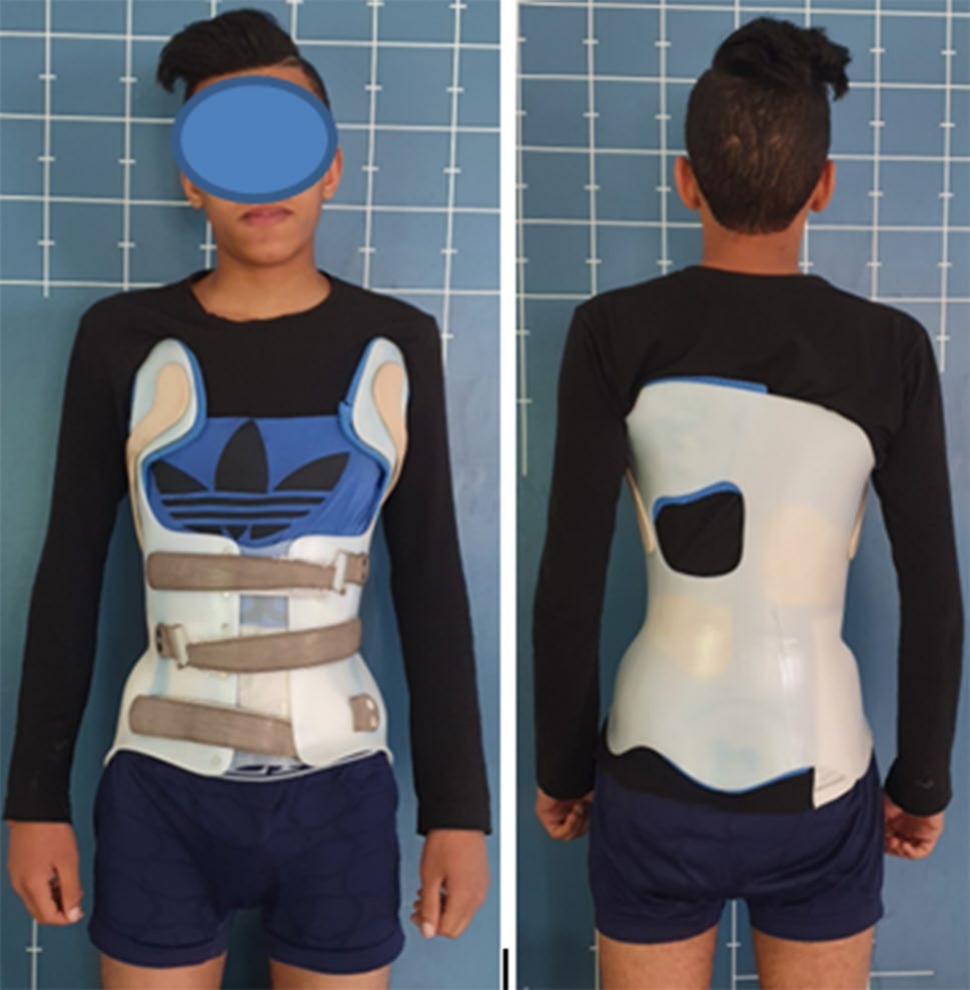
CTM corset in place for 1 year

**Fig. 7:**
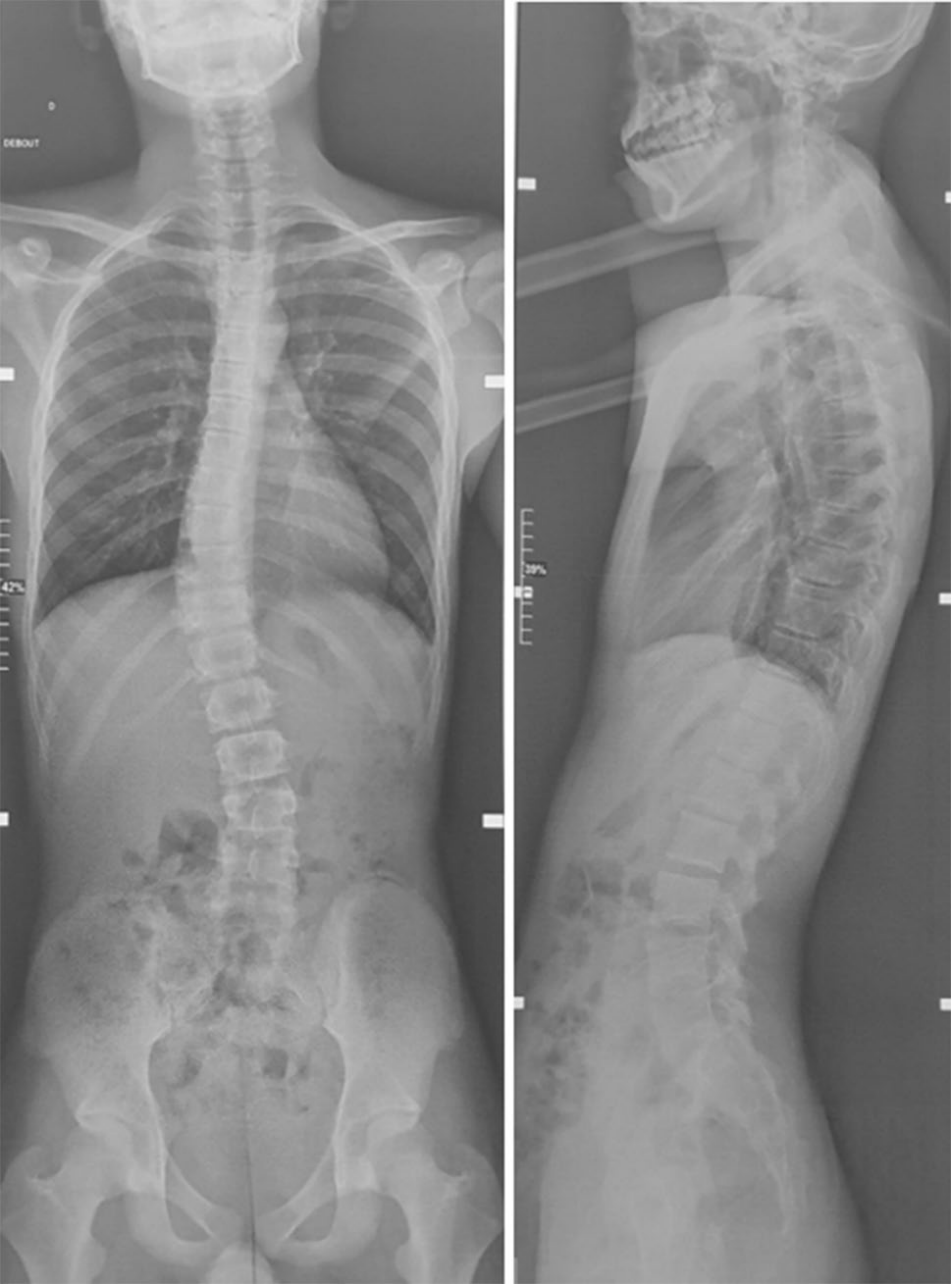
Radiological control at 3 years: correction of scoliosis with a residual Cobb angle of 26°

We report the case of a 14-year-old patient who consulted for spinal deformity. The patient reported nocturnal spinal pain which had progressed for 5 months. When standing, the shoulders were horizontal, the line of the thorns was deviated in italic “S” to the right (Fig. 1). Leaning forward, there was a right thoracolumbar gibbosity (Fig. 1). Stiffness of the spine associated with contracture of the paravertebral muscles. The neurological examination was without abnormality. The patient received medical treatment with salicylates. We noted a disappearance of nocturnal pain. An X-ray examination revealed thoracolumbar scoliosis. The Cobb angle from T7 to L2 was 52° (Fig. 1). Bone scintigraphy showed hyperfixation of the left superior articular process of T11 (Fig. 2). A CT scan concluded with a rosette image with a central nidus suggestive of OO (Fig. 3). MRI showed signal abnormalities in the left superior articular process of T11, an intense response of peripheral soft tissues (Fig. 4). The therapeutic strategy was the resection of the OO by a minimally invasive posterior approach to the spine. The histological study confirmed the diagnosis of OO. The management of scoliosis was a non-operative treatment. A corset plastered on the traction framework according to Cotrel was performed to correct the scoliosis (Fig. 5). This immobilization is kept for 3 months. The relay is provided by a CTM brace for 1 year (Fig. 6). The clinical course was marked by the disappearance of nocturnal pain and a correction of the spinal deformity. At 3 years, the radiological control confirmed the improvement of the Cobb angle from 52 to 26° (Fig. 7).

## Discussion

OO is a benign bone lesion with high incidence at the age between 10 and 20 [1]. It occurs more in extremities than in spine [2, 3]. The lumbar spine is the most commonly affected area, followed by the cervical, thoracic, and sacral regions [4]. The diagnosis of OO is primarily clinical. In typical cases, it is characterized by inflammatory pain at night [2, 5–7]. Nonsteroidal anti-inflammatory drugs (NSAIDs) are used to relieve pain [5]. This is a diagnostic test. The pain is due to irritation of the nerve fibers in contact with the nidus [3] and the peritumoral inflammatory reaction due to prostaglandins (cyclooxygenase-2) [6]. Scoliosis is mainly due to muscle spasms and chronic inflammatory reactions surrounding the tumor. [4, 5]. If the diagnosis is late, a structured scoliosis forms with spinal rotation [8]. It has been demonstrated that for the spinal location of OO, the diagnostic delay can reach 54% in some series [9]. The X-ray of the entire spine makes it possible to study the frontal and sagittal balance of the spine, in particular to classify scoliosis and to objectify spinal rotation (structured scoliosis). CT allows a good study of OO by objectifying the nidus centered by calcifications and peripheral bone condensation [10]. Bone scintigraphy allows precise localization of the lesion [6]. MRI is an essential adjunct. It makes it possible to eliminate a malignant tissue process, to check that there is no spinal or root compression and to objectify the peri-lesional edema. Surgical treatment consists of resection of the entire tumor, preferably in one piece [5], to ensure healing without risk of recurrence. Treatment of spinal OO can also be done by radiofrequency [11, 12], but the risk of neurological damage is high, particularly for locations in the posterior column. This should remain a contraindication until further notice [13]. For the management of scoliosis, when it is non-structural, the deformity spontaneously reduces after resection of the OO and the disappearance of local inflammatory signs. The problem arises with structured scoliosis. In this case, we recommend a non-operative treatment to preserve spinal mobility. But in case, resection of the articular process causes spinal instability, treatment with posterior instrumentation and fusion becomes a potential treatment option [2].

## Conclusion

We encountered an unusual case of OO of the thoracic spine presenting as scoliosis that was difficult to diagnose. If the OO nidus exhibits close proximity to the neural canal, resection of the tumor is an effective and safe treatment method. The duration of symptoms and the age at the presentation time are the most important factors in the development of associated vertebral rotation, a structural scoliosis with a high magnitude of curve.

## Data Availability

All data are available for the reading committee.
